# Strain-Driven Honeycomb Reconstruction of Multilayered Rh/Pt(111)

**DOI:** 10.1021/acsami.5c25647

**Published:** 2026-05-25

**Authors:** Abdulla Bin Afif, Oleksii Ivashenko, Alexandra Jahr Kolstad, Anja Olafsen Sjåstad

**Affiliations:** † Centre for Materials Science and Nanotechnology, Department of Chemistry, 6305 University of Oslo, P.O. Box 1033 Blindern, N-0315 Oslo, Norway; ‡ 67034 DNV AS, Veritasveien 1, N-1363 Høvik, Norway

**Keywords:** Scanning tunneling microscopy, Honeycomb network, Strain-driven surface reconstruction, Atomic diffusion, In situ X-ray photoelectron spectroscopy, Low-energy electron diffraction, Pt−Rh catalyst

## Abstract

This study reports on a long-range ordered honeycomb surface structure formed by annealing of Rh multilayers on Pt(111). Scanning tunneling microscopy reveals a honeycomb network with a lateral periodicity of ∼15 nm and ∼0.07 nm corrugation, most pronounced at ∼11.2 ML Rh coverage and annealing at 700 K. In situ X-ray photoelectron spectroscopy at 700 K, combined with diffusion calculations, indicates Pt surface enrichment and Pt–Rh intermixing driven by surface and grain boundary diffusion, while bulk diffusion remains negligible. At 900 K, bulk diffusion becomes dominant, and the honeycomb structure is disrupted upon prolonged annealing. The honeycombs arise from an interplay among Rh coverage, annealing time and temperature, and Pt–Rh mixing dynamics and are driven by interfacial strain due to lattice mismatch, which relaxes through surface reorganization. These findings highlight the delicate interplay of atomic diffusion and strain-driven reconstruction in bimetallic nanostructure evolution.

## Graphical abstract

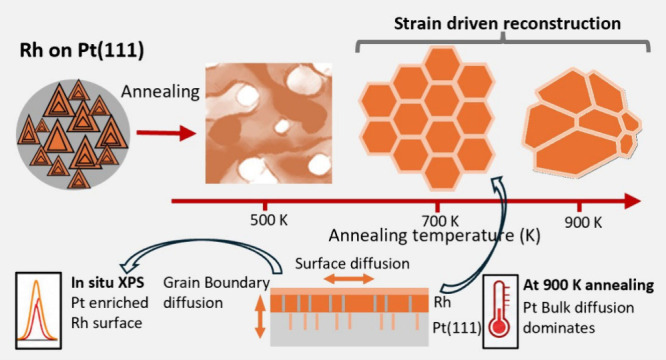

## 1 Introduction

Platinum–rhodium (Pt–Rh) based alloys are essential for many industrial applications due to their superior catalytic performance. Key examples include the Ostwald process for nitric acid (HNO_3_) production,^1^ the Andrussow oxidation for hydrogen cyanide (HCN) synthesis,^2^ low temperature polymer electrolyte membrane fuel cells (PEMFCs), and emission-control applications such as NOx reduction, NH_3_ (slip) oxidation, and CO oxidation. Industrial HNO_3_ and HCN production typically take place at elevated temperatures over expensive Pt–Rh gauges. In contrast, for processes operating at milder temperatures, supported nanoparticles offer an attractive and more economical alternative. In the maritime sector, increasing interest in NH_3_-fueled engines has highlighted the need for effective NH_3_ slip mitigation,^3^ with selective oxidation of NH_3_ over supported Pt–Rh nanoparticles as a promising abatement technology.

Bi- and multicomponent metallic systems are structurally complex and may undergo significant restructuring, segregation or oxidation under reaction conditions. This dynamic behavior makes it difficult to unambiguously identify active sites and dominant reaction pathways when used as catalysts. Therefore, systematic studies of well-defined, alloyed single crystals using (in situ/operando) surface science techniques [X-ray photoelectron spectroscopy (XPS), scanning tunneling microscopy (STM), surface X-ray diffraction (SXRD), low energy electron diffraction (LEED) etc.] are essential to reveal the surface’s chemical state and atomic structure. These insights guide the design of more active, selective, and stable catalysts.

Studies of well-defined Pt–Rh model surface catalysts for the selective NH_3_ oxidation to N_2_ under NH_3_ slip conditions^4^ indicate that product distribution (N_2_, N_2_O, NO) depends on Pt–Rh surface element distribution, process conditions, and nanoscale populations of adsorbed N- and O-species. In previous work,^5,6^ mapping of conditions for the formation of Pt–Rh surface structures involving deposition and annealing of low-coverage (<1 ML) Rh on Pt(111) and Pt on Rh(111) was studied using STM and LEED to monitor structural transitions. Building on this foundation, the present work introduces an extended roadmap (Section 3.8) that incorporates the high Rh coverage regime and the newly identified long-range ordered honeycomb reconstruction.

Surface reconstruction is a common mechanism for strain relief in metals and alloys. Although metals and alloys can display similar surface patterns, their origins differ. Intrinsic surface stress drives reconstruction in metals, whereas in alloys they result from compositional segregation and lattice mismatch. Au(111) and Pt(111) are classic examples, where Au forms a herringbone pattern, while Pt(111) exhibits sparse stripe networks. Furthermore, Pt(111) is intrinsically sensitive to environmental conditions, undergoing transformations into honeycomb or triangular motifs.^7^ These reconstructions are characteristic of 5d metals such as Pt, Ir, and Au but are absent in 4d metals such as Pd and Rh.^8^ In contrast, for both Pt_3_Sn­(111)^9^ and Pt_25_Ni_75_(111),^10^ honeycomb patterns appears from surface reconstruction driven by lattice mismatch in the top atomic layers induced by a preferential sputtering and annealing. A similar strain-relief mechanism operates in epitaxially grown CoSi_2_ on Si(111),^11^ where honeycomb patterns form above a critical CoSi_2_ thickness. For Pt_3_Sn­(111), preferential sputtering depletes Sn relative to Pt, causing Sn diffusion to the topmost layer during annealing at intermediate temperatures (700–800 K). This process induces tensile stress in the Sn-depleted subsurface region, where the lattice parameter approaches that of monometallic platinum (*a*_Pt_ = 3.92 Å^9^), which is smaller than bulk Pt_3_Sn (*a*_Pt_3_Sn_ ∼ 4.00 Å).^12,13^ Simultaneously, the Sn-enriched top layer becomes compressively strained. The resulting lattice mismatch is relieved by subsurface misfit dislocations, producing a honeycomb network characterized by (√3 × √3)­R30° domains with a mean width of ∼15 nm (diagonal length of ∼17.3 nm) and a height modulation of 0.06–0.07 nm. A related mechanism is proposed for Pt_25_Ni_75_(111),^10^ where preferential sputtering and postannealing lead to Pt enrichment near the surface. This increases the local lattice constant relative to the bulk Pt_25_Ni_75_ alloy (*a*_Pt_ = 3.92 Å^9^ and *a*_Ni_ = 3.52 Å^14^), generating lattice mismatch and subsurface misfit dislocations that drive honeycomb formation.

Honeycomb structures are not limited to vacuum-annealed alloy systems but also form under specific chemical conditions and in distinct material systems. For example, atomic-resolution imaging has revealed honeycomb formation during the electrochemical transformation of Ni on Pt(111) in alkaline media under hydrogen evolution reaction conditions.^15^ A similar motif has also been observed in a nonmetallic overlayer, namely an epitaxial Nb_2_O_3_ honeycomb monolayer formed on Au(111) by metal deposition followed by oxidation.^16^

This study investigates how thicker heteroepitaxial Rh films (≤11.2 ML) on Pt(111) relieves stress by the formation of a long-range ordered honeycomb surface structure. The structure emerges upon postdeposition annealing at 700 K, as evidenced by STM. The honeycomb network is well-defined, consistently observed across different tunneling parameters, and extends across atomic steps. Its formation likely arises from intermixing and alloying in the top Rh surface, Pt interdiffusion from the bulk along Rh grain boundaries during thermal processing, subsequent lateral surface diffusion of Pt and Pt–Rh species, and defect-driven strain relaxation due to lattice mismatch between the Pt–Rh top layer and Rh rich subsurface. The proposed mechanism is supported by experimental findings obtained from STM, LEED, in situ XPS and coupled to diffusion properties of Pt and Rh in bulk, grain boundaries and surfaces.^17–19^

## 2 Experimental Section

### 2.1 General Description of Multipurpose Surface Science Equipment

Experiments were conducted using a commercial STM system developed by Leiden probe microscopy B.V.^20^ This system consists of three main interconnected chambers: a preparation, an XPS, and a STM chamber, all maintaining a base pressure of 3–5 × 10^–10^ mbar. This ensures fully inert handling of the samples through sample preparation, XPS analysis and STM scanning. The preparation chamber facilitates standard sample treatments, including Ar^+^ ion sputtering (IQE 11–35, SPECS), thermal annealing (up to 1300 K), metal deposition using a four-pocket electron beam (e-beam) evaporator (EBE-4, SPECS), and gas dosing through leak valves. It also includes equipment for low-energy electron diffraction (ErLEED 3000D, SPECS) to verify crystalline structure. The XPS chamber is equipped with a SPECS PHOIBOS 150 1D-DLD energy analyzer, a monochromatic focus 500 X-ray source, and the sample stage operating up to 1300 K. The STM chamber allows imaging in UHV and in a small (mL), high pressure flow cell in a dynamic gas mixture up to 6 bar.

### 2.2 Sample Preparation

The Pt(111) single crystal (99.999%; surface preparation laboratory (SPL), Netherlands) was cleaned through alternating cycles of Ar^+^ sputtering and annealing. Sputtering was performed using 1 kV Ar^+^ ions at a pressure of 1.1 × 10^–5^ mbar for 10 min, followed by annealing at 1100 K under UHV conditions for 10 min. Typically, 5–7 cleaning cycles were carried out prior to Rh deposition. For the highest Rh coverage used in this study (11.2 ML), removal of the overlayer by 7 cycles of sputtering and annealing was followed by XPS, showing no detectable Rh signals in the XPS spectra [Supporting Information (SI), Section S1].

For each experimental condition a freshly cleaned Pt(111) surface at 350 K was used, on which Rh (99.9%, Goodfellow) was evaporated using an e-beam evaporator. The deposition was performed with an e-beam flux of 8 nA, yielding a surface coverage rate of ∼0.35 ML/min, for durations ranging from 4 to 32 min, as inferred from STM data.

Thermal behavior of the samples was studied using samples initially prepared at 350 K, followed by postdeposition annealing at 500, 700, and 900 K for 6–32 min, with each annealing temperature tested on a separate, freshly prepared surface.

For accurate temperature monitoring, particularly above 900 K, a K-type thermocouple was used in combination with a Micro-Epsilon TIM 1 M infrared thermal camera, calibrated with an emissivity setting of 0.85 for polished Pt surfaces.

### 2.3 Scanning Tunneling Microscopy (STM) and Data Analysis

STM imaging was conducted using Pt_80_Ir_20_ tips (Goodfellow, 0.25 mm diameter) operated in constant current mode under UHV conditions, controlled by the CAMERA 4.3 software package from Leiden University. Imaging parameters included a sample bias ranging from −0.5 to −1 V and a tunneling current of 0.1 nA.

Surface features such as coverage and layer height were analyzed from STM images using the Gwyddion software package.^21^ Horizontal scars were corrected, and a polynomial background subtraction was applied. Adlayer coverage was evaluated using a threshold height method, excluding areas near step edges. Due to the similar atomic densities of Pt and Rh, Rh overlayer coverage was calibrated based on the projected area of Rh layers. WSxM 5.0 software was utilized for STM image processing.^22^

The Rh surface coverage was calculated for the film obtained at the shortest deposition time (4 min) by summing the Rh contributions from all observed layers (SI, Section S2). Heights corresponding to a single atomic layer of Rh were identified using thresholds of 0.22 nm. For thicker films (up to ∼11.2 ML), the total coverage was estimated from the calibrated evaporation rate (at 4 min) as not all layers can be directly resolved in STM.

### 2.4 X-ray Photoelectron Spectroscopy (XPS) and Data Analysis

XPS measurements were carried out using a monochromatic Al Kα X-ray source (1486.6 eV) positioned at a 54° angle relative to the surface normal. Photoelectrons were detected along the surface normally using an analyzer set with a pass energy of 10 eV and a dwell time of 0.1 s. The electron spectrometer and data acquisition were managed through the SpecsLab Prodigy software (SPECS GmbH). All reported binding energy values (BEs) are reported with an uncertainty of ±0.1 eV.

In situ XPS was employed to monitor Pt–Rh intermixing and alloy formation during the thermally induced development of the honeycomb surface structure. E-beam heating was used to achieve and sustain the temperature between 350 and 700 K under ultrahigh vacuum conditions. XPS spectra were continuously acquired throughout the annealing process, enabling real-time tracking of chemical state changes and surface composition evolution.

### 2.5 Low-Energy Electron Diffraction (LEED)

Surface structures were examined using LEED (ErLEED from SPECS). The cathode was operated at 2.3 A and the fluorescent screen at 5000 V. E-beam energy was varied from 0 to 200 eV, and LEED patterns were captured using a phone camera.

## 3 Results and Discussion

### 3.1 Morphology of Thick Rh/Pt(111) as Prepared

The growth of Rh islands prepared on Pt(111) at 350 K was investigated using STM imaging. Figure 1a shows uniformly distributed and consistently aligned triangular islands across the flat terraces of the Pt(111) with a calculated surface coverage of 1.4 ML (SI, Section S2). The measured step heights of ∼0.21 nm align with the reported monolayer thickness of Rh.^5^ Line profile analysis (Figure 1b) reveals a height of approximately 0.65 nm, consistent with three Rh atomic layers, confirming a three-dimensional growth mode at the applied conditions, and consistent with previous studies on Rh/Pt(111),^5,6^ Pt/Pt(111),^23,24^ and Ru/Pt(111)^25^.

**Figure 1:**
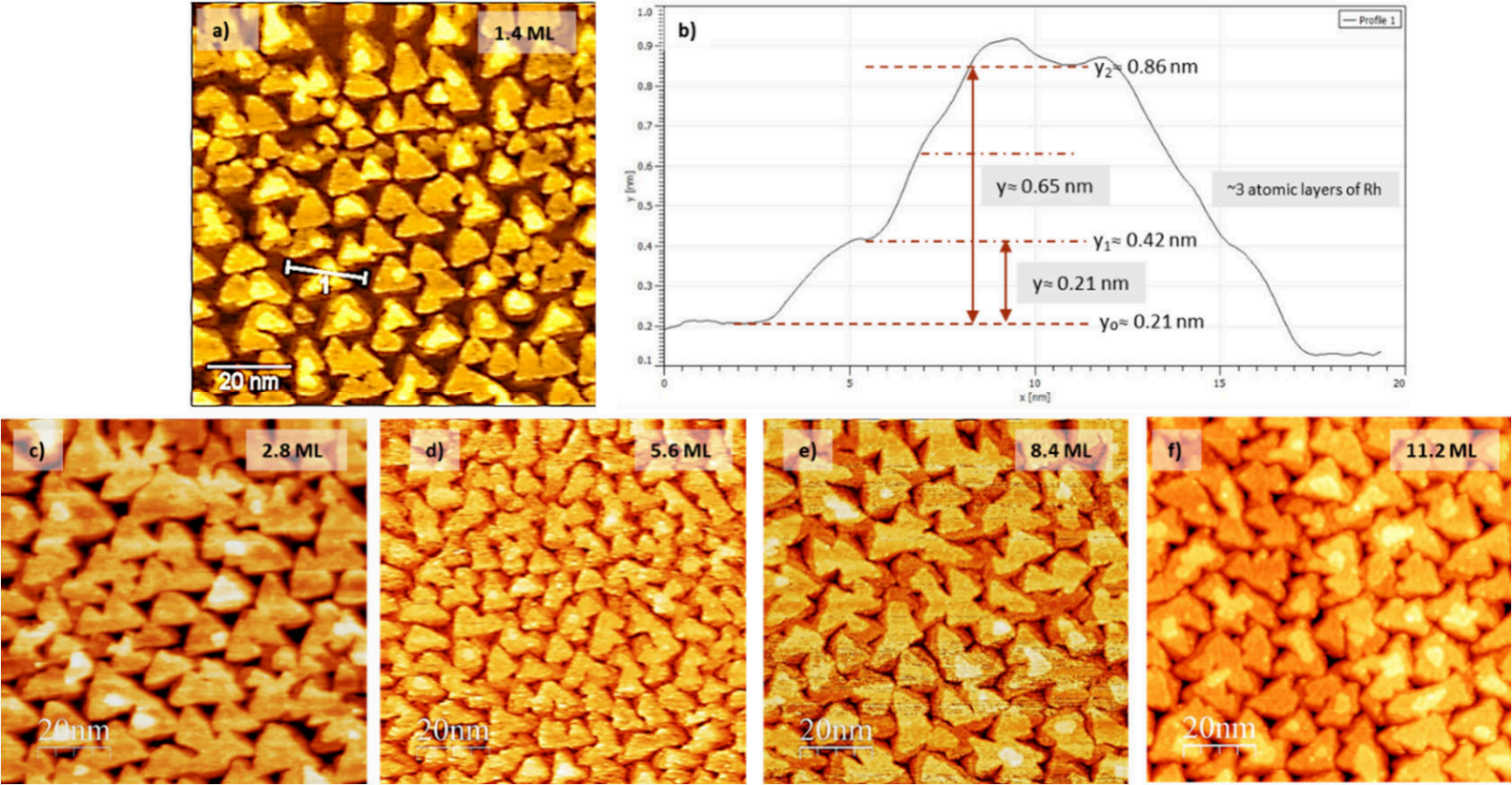
(a,b) STM topography and line profile analysis of 1.4 ML Rh deposition on Pt(111) at 350 K, showing uniformly distributed triangular structures aligned in the same orientation. (c) At 2.8 ML, islands enlarge, and coverage increases with some voids remaining. (d) At 5.6 ML, the surface is nearly fully covered with closely packed islands. (e,f) At 8.4 and 11.2 ML, coalesced island structure.

The evolution of surface morphology with estimated Rh coverage of 2.8 ML, 5.6 ML, 8.4 ML, and 11.2 ML are shown in Figure 1c–f. At 2.8 ML, triangular islands grow larger, and they are more densely populated than the 1.4 ML surface, though voids remain. By 5.6 ML, further islands coalescence results in near-complete surface coverage. At 8.4 and 11.2 ML, islands grow even larger and stack into secondary layers, while their triangular shape and alignment remain preserved, indicating a persistent growth mode and surface ordering.

### 3.2 Formation of Long-Range Ordered Honeycomb Structure during Annealing

When subjecting the as-deposited Rh/Pt(111) surfaces with Rh coverage between 5.6 and 11.2 ML to 700 K for 24 min a honeycomb surface reconstruction appears, although to varying extent. For the 5.6 ML sample (Figure 2a), STM shows that the reconstruction is incomplete across the terraces, despite Rh coverage is substantial. Height variations and uneven top-layer morphology across the terraces, suggest local differences in the degree of Pt–Rh mixing or strain, hindering the honeycomb formation propagating uniformly.

**Figure 2:**
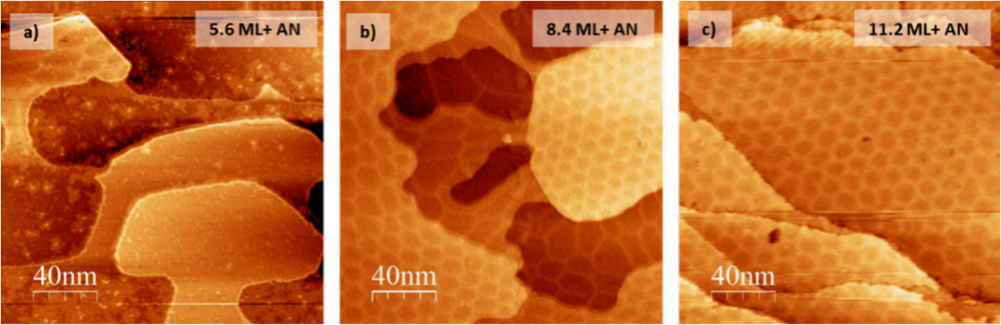
STM topography of surface reconstruction forming as a result of increased Rh coverage on Pt(111), after postdeposition annealing at 700 K for 24 min. (a) At 5.6 ML, localized growth of a honeycomb pattern begins to emerge in the top-left corner. (b) At 8.4 ML, a honeycomb structure forms on top layers, and there is incomplete reconstruction in the deeper layers. (c) At 11.2 ML, a well-defined and uniform honeycomb structure is observed across the surface.

At 8.4 ML (Figure 2b), the honeycomb structure becomes more apparent across the surface. Despite the reconstruction is more developed, the morphology is still nonuniform. In contrast, the 11.2 ML sample (Figure 2c), exhibits a well-defined and uniform honeycomb structure throughout the entire surface. This observation highlights the critical role of initial Rh coverage and mixing of Pt and Rh in enabling the structural transformation necessary for the formation of a well-defined and uniform honeycomb structure across the entire surface.

### 3.3 Effect of Postdeposition Annealing Time and Temperature

The effect of postdeposition annealing time on honeycomb formation was studied by annealing at 700 K for 6, 12, 24, and 30 min. For each annealing time, 11.2 ML Rh was deposited at 350 K on a freshly cleaned Pt(111) surface to ensure consistent conditions.

After 6 min annealing, Rh/Pt(111) shows a highly uniform and well-defined honeycomb pattern (Figure 3a), suggesting rapid atomic rearrangement at 700 K. The individual hexagonal cells exhibit a diagonal (corner-to-corner) length of ∼14.6 ± 1.7 nm (SI, Section S3) and feature height of 0.07 nm (Figure 3b). It was observed that prolonging the annealing to 30 min does not significantly change the honeycomb size or shape (SI, Sections S3 and S4).

**Figure 3:**
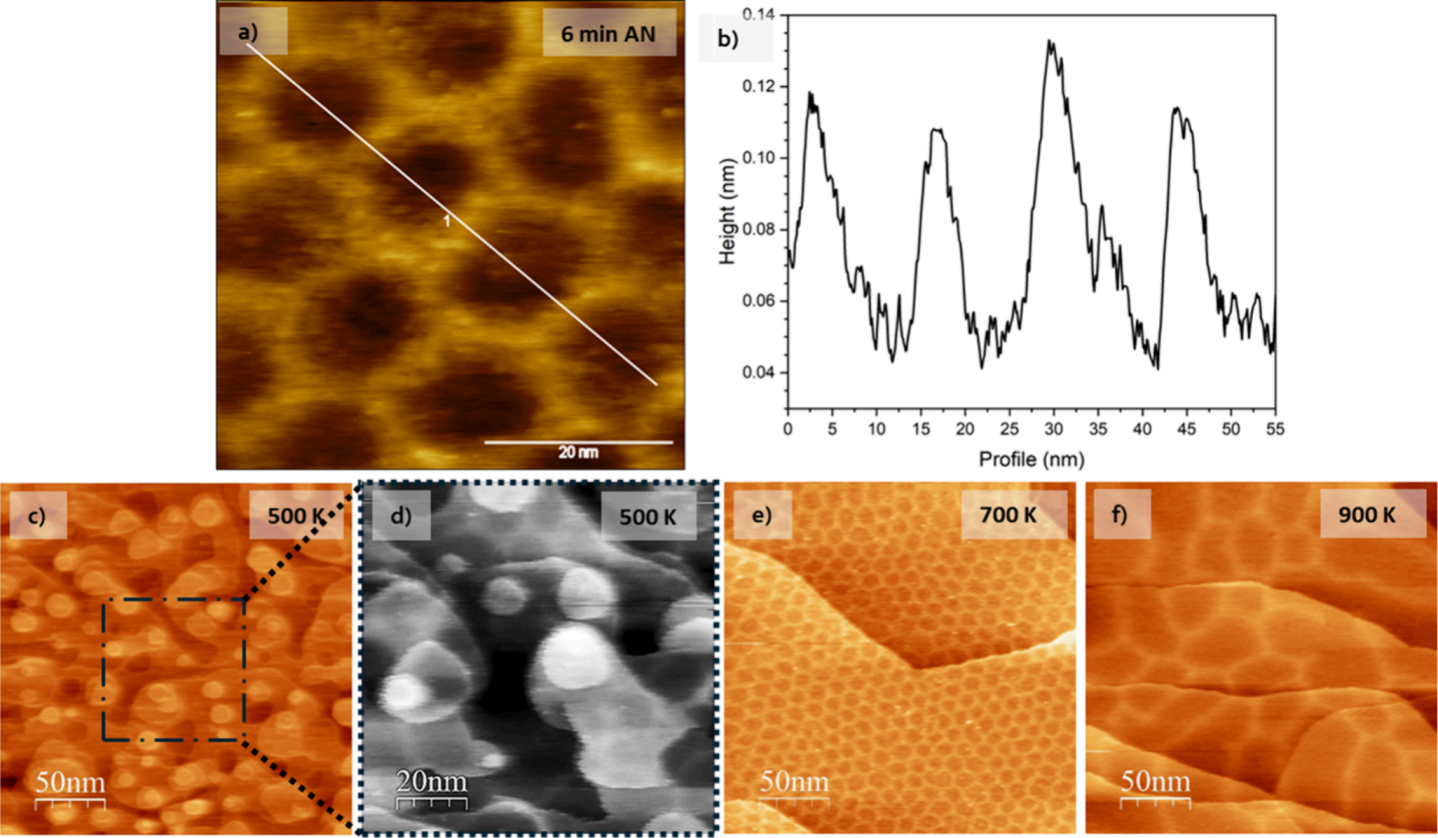
STM images of Rh/Pt(111) surfaces after deposition of 11.2 ML Rh at 350 K followed by postdeposition annealing. (a) STM image after 6 min annealing at 700 K, showing a well-defined honeycomb pattern. (b) Corresponding line profile (indicated in a) reveals a corrugation of ∼0.07 ± 0.01 nm. (c) STM image after 24 min annealing at 500 K, showing a porous Rh film with worm-like features and small islands, indicating incomplete reconstruction. (d) Higher magnification image (20 nm scale) highlighting granular clusters and early stages of honeycomb-like ordering (position in c is representative). (e) After 24 min annealing at 700 K, a well-defined and continuous honeycomb structure is observed. (f) After 24 min annealing at 900 K, extensive surface restructuring leads to distorted and elongated honeycomb features, along with step bunching due to enhanced atomic mobility.

The effect of postdeposition annealing temperature (500–900 K) on Rh/Pt(111) was studied by depositing 11.2 ML Rh at 350 K on a fresh surface, followed by 24 min of annealing for each condition. STM images (Figure 3c–f) reveal clear temperature-dependent changes in the surface structure.

At 500 K (Figure 3c), the surface shows a porous, worm-like morphology with small circular islands or agglomerates, while higher magnification (Figure 3d) reveals traces of honeycomb ordering. This indicates that 500 K is adequate for initiating pattern formation but is insufficient for the full restructuring into a uniform flattened top layer within 24 min of annealing time. The limited reconstruction is attributed to a diffusion-limited effect. At this lower temperature, Pt diffusion from the substrate to the surface is expected to be low (Section 3.6), delaying the formation of a Pt–Rh enriched top layer that drives long-range ordering.

Annealing at 700 K for 24 min (Figure 3e) produces a uniform and well-ordered honeycomb surface structure, indicating optimal conditions for atomic diffusion and rearrangement. Interestingly, by annealing at 900 K for 24 min, the honeycombs become distorted, with larger, irregular hexagons (Figure 3f), similar to patches observed at lower coverages annealed at 700 K (Figure 2a,b). Shorter postdeposition annealing, 6 min at 900 K (SI, Section S5), yields regular honeycomb patterns comparable to 24 min at 700 K (Figure 3e). Diffusion calculations (Section 3.6) show bulk Pt–Rh mixing increases dramatically from 700 to 900 K, promoting Pt surface enrichment due to its lower surface energy relative to Rh.^26,27^ This disrupt the stoichiometry and strain conditions necessary to stabilize the fine periodicity, explaining the irregular hexagons. The irregular hexagons observed in Figure 2a,b can be explained by a similar argument. Irregular hexagons of this kind are also reported for bare Pt(111) surfaces.^28^

Based on the temperature intervals explored in this study (500, 700, and 900 K), our results indicate that long-range ordered honeycomb surface reconstructions can be reliably achieved within a thermal window centered around 700 K for a 24 min postdeposition annealing step. Importantly, the same well-defined uniform honeycomb reconstruction can be obtained at higher temperatures, such as 900 K with shorter annealing (e.g., 6 min; see SI, Section S5), or potentially at lower temperatures than 700 K with extended postdeposition annealing (>24 min). This observation indicates temperature and annealing time and thus interplay to control both the Pt–Rh intermixing kinetics during formation and the stability of the resulting periodic surface morphology.

To determine the structural origin of the long-range order, we evaluated whether the hexagonal honeycomb pattern could be attributed to a moiré superstructure. This evaluation involved a comparative analysis of the Fast Fourier Transform (FFT) derived from the experimental honeycomb pattern obtained after annealing at 900 K for 6 min and compared with a simulated FFT of a relaxed Pt(111) overlayer on Rh(111) (SI, Section S6.1). The FFT of the honeycomb structure displays a streak through the origin, rather than a single, small, symmetric hexagonal spot pattern characteristic of a simple geometric moiré. For comparison, we also include simulated real-space images and corresponding FFTs of twisted graphene bilayers, which exhibit well-defined moiré signatures with discrete hexagonal spot sets.^29^ This comparison highlights that, while the honeycomb structure exhibits long-range order, its FFT characteristics are inconsistent with those of a moiré system.

To further assess whether the observed ∼15 nm periodicity could arise from a simple geometric moiré, we estimated the expected moiré length for a Pt(111) overlayer on Rh(111) using representative lattice parameters (SI, Section S6.2). For a fully relaxed Pt(111) surface (*a*_Pt_ = 3.99 Å)^30^ on Rh(111) (*a*_Rh_ = 3.80 Å), the calculated moiré periodicity is approximately 10 nm, which is significantly smaller than the experimentally observed Pt–Rh honeycombs. Using the bulk Pt lattice constant (*a*_Pt bulk_ = 3.92 Å)^9^ yields a moiré periodicity of ∼15 nm; however, our in situ XPS data (Section 3.5) do not support formation of a pure surface Pt overlayer, whereas assuming a reduced Pt lattice parameter representative of a Pt–Rh alloyed surface (e.g., *a*_Pt–Rh_ = 3.85 Å) results in an even larger moiré length (∼40 nm). These comparisons indicate that the observed honeycomb spacing cannot be consistently explained by a purely geometric moiré between rigid lattices.

Additional support for a nonmoiré origin comes from annealing at 900 K for longer durations (24 min, Figure 3f), which leads to distorted and irregular honeycomb networks. Moreover, moiré patterns are typically highly ordered and periodic, whereas the STM images reveal locally distorted, elongated, and nonuniform honeycomb units at elevated temperatures. This spatial variability is incompatible with rigid-lattice interference and instead supports a strain-driven surface reconstruction mechanism.

### 3.4 Surface Structures of Pt(111), As-Deposited and Postannealed 11.2 ML Rh/Pt(111)

LEED was conducted to explore structural changes in the topmost atomic layers of 11.2 ML Rh/Pt(111) during honeycomb structure formation. Initial measurements on a freshly cleaned Pt(111) surface featured six diffraction spots with a hexagonal symmetry (Figure 4a), consistent with the Pt(111) surface.^31^ This surface served as the baseline for subsequent experiments.

**Figure 4:**
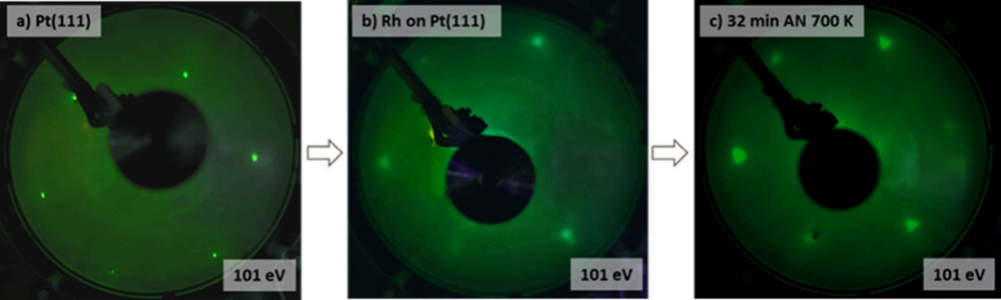
LEED patterns of (a) freshly cleaned Pt(111) showing six diffraction spots with similar intensity in the diffraction spots in the hexagonal symmetry, (b) after deposition of 11.2 ML Rh on Pt(111) at 350 K resulting in a characteristic Rh(111) pattern (three intense and three less intense diffraction spots in the hexagonal symmetry) but with broader spots, and (c) 11.2 ML Rh/Pt(111) annealed at 700 K showing triangular composite Rh(111) diffraction spots, signatures of enhanced ordering and domain coexistence in the honeycomb reconstruction.

After deposition of 11.2 ML Rh on Pt(111) at 350 K, LEED displayed three strong and three weaker diffraction spots (Figure 4b), characteristic of the Rh(111) surface.^32^ Notably, the diffraction spots are diffuse. Following postdeposition annealing at 700 K, we note that the three pronounced diffraction spots exhibit a distinct triangular shape (Figure 4c). The spot profile (shape, width, and energy dependence) in LEED is an indicator of long-range order, domain size, and surface morphology.^33^ The triangular shape likely results from the coexistence of three rotationally equivalent domains of the Rh layer below Pt enriched Rh top layer.^34^ We interpret these features not as single Bragg reflections, but as composite diffraction spots representing multiple, closely spaced and unresolved spots. This composite structure may be used as an indicator of the honeycomb reconstruction observed in Figure 3e.

LEED results indicate that annealing facilitates self-organization of the top atomic layers into a flatter, and more ordered arrangement via enhanced surface mobility at elevated temperatures. The LEED pattern of the honeycomb surface structure retains an overall hexagonal symmetry similar to Rh(111), but the diffraction spots exhibit a distinct triangular composite shape. No resolvable satellite spots are observed, which is consistent with the large real-space periodicity (∼15–17 nm) of the honeycomb structure placing the corresponding reciprocal-space splitting below the LEED resolution.^35^

### 3.5 In Situ XPS during Annealing of Rh on Pt(111)

In situ XPS was used to investigate Pt–Rh intermixing/alloying during formation of the honeycomb structure at 700 K. Two identical experiments were carried out, both investigating 11.2 ML Rh deposited on Pt(111), followed by annealing at 700 K for approximately 32 min, while XPS spectra for Rh 3d (experiment 1) and Pt 4f (experiment 2) were continuously collected throughout the process (Figures 5 and 6).

**Figure 5:**
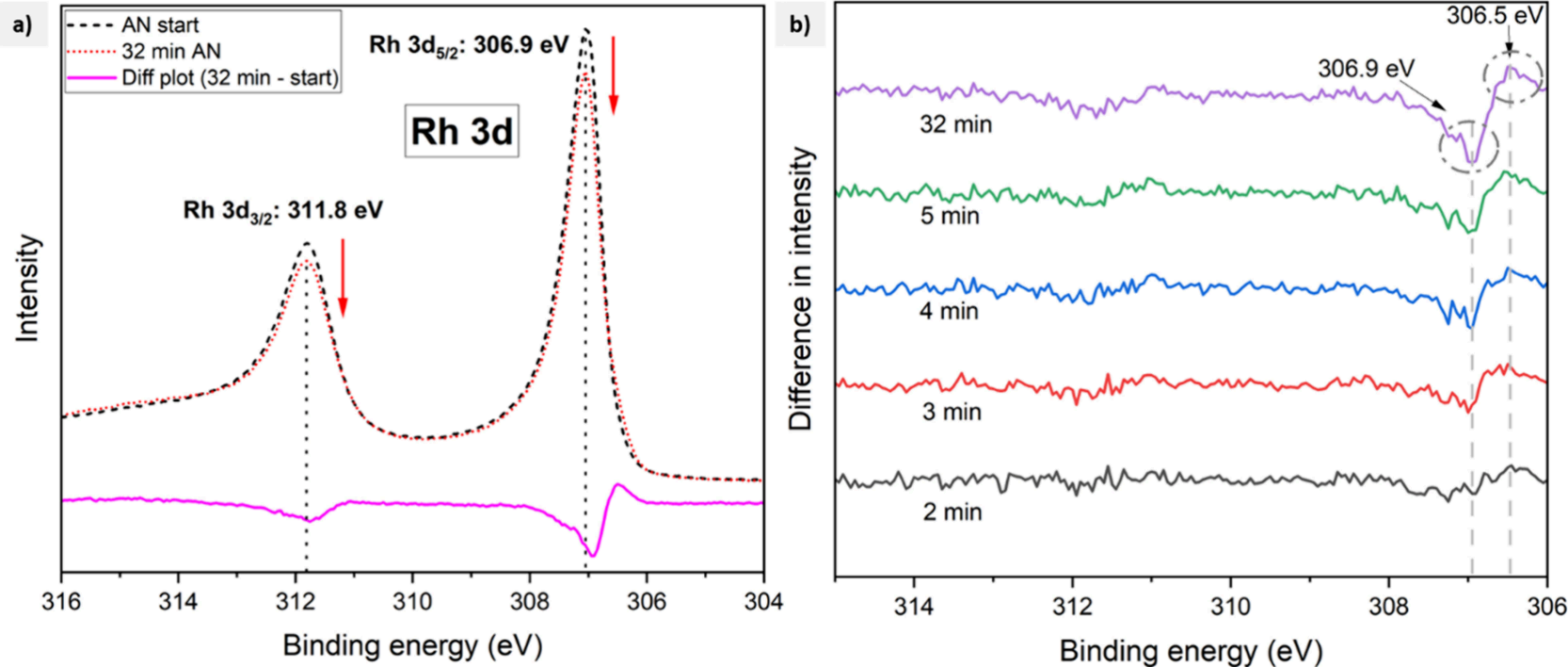
In situ XPS analysis of the Rh 3d core level during postdeposition annealing of 11.2 ML Rh on Pt(111) at 700 K. (a) Difference between as prepared and after 32 min of annealing. (b) Difference spectra (*t* – *t*_0 min_ = 2, 3, 4, 5, and 32 min) of the Rh 3d region, highlighting the emergence of a new component at ∼306.9 eV within the first 3–4 min. This feature remains stable in shape and intensity throughout the experiment, accompanied by a concurrent decrease in the component at ∼307 eV, indicating the development of a chemically distinct Rh environment.

**Figure 6:**
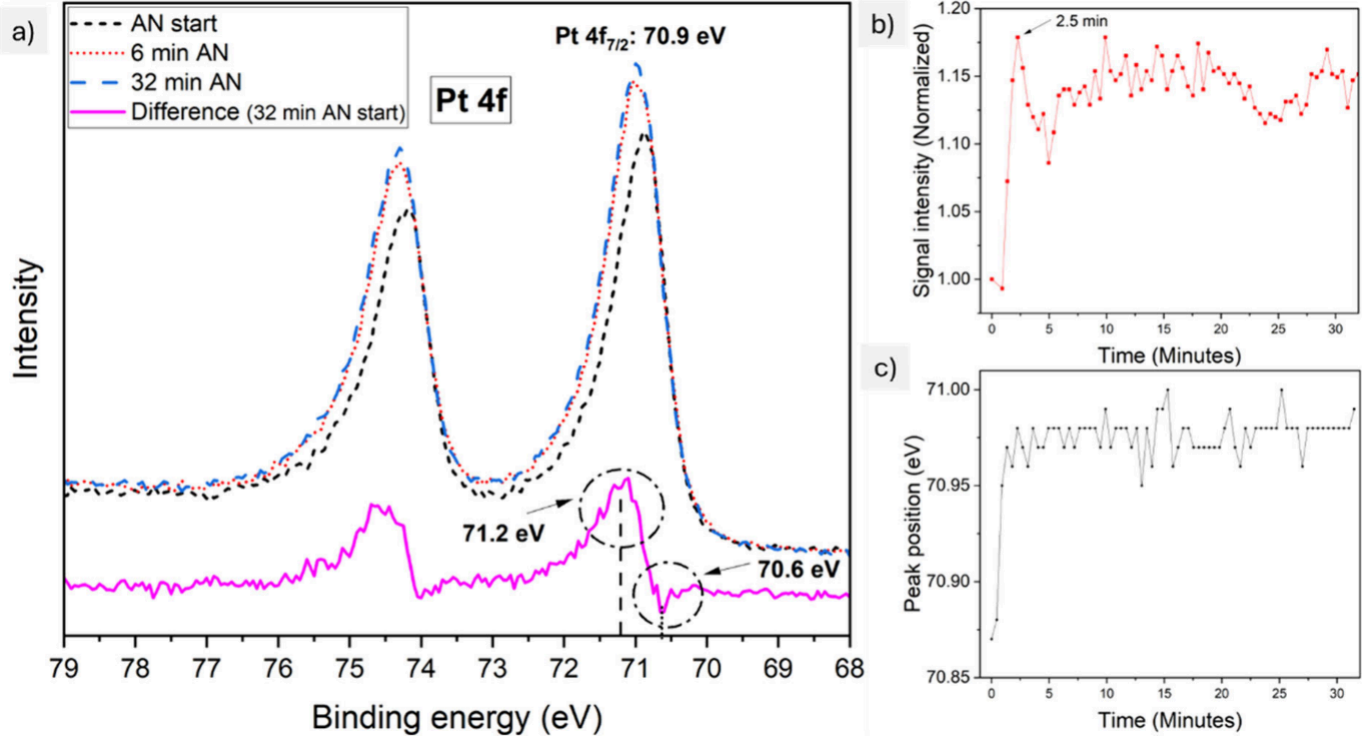
(a) In situ XPS spectra of the Pt 4f core level acquired before annealing and after 6 and 32 min postdeposition annealing at 700 K. An increase in intensity and an ∼0.1 eV shift is observed, indicating a new chemical state emerging for Pt. The difference spectrum (*t*_32 min_ – *t*_0 min_) highlights the net gain in Pt signal, particularly on the low-binding energy side. (b) Time evolution of the normalized Pt 4f_7/2_ peak intensity (relative to the first measurement) during annealing at 700 K. (c) Corresponding shift in the Pt 4f_7/2_ binding energy position over the same period.

The XPS data collected in the first experiment revealed a reduction in intensity of the Rh 3d components after 32 min of annealing (Figure 5a). The observed reduction in the Rh 3d intensity indicates less Rh present within the analyzed volume, pointing to either Rh going into the subsurface region or transport of Pt to the surface, or a combination of both.

To understand better the dynamics in surface composition during annealing, difference spectra were produced by subtracting the initial (*t* = 0 min) Rh 3d spectrum from those recorded at *t* = 2–32 min, as shown in Figure 5b. These difference spectra reveal progressive changes in the surface chemical environment induced by annealing. After 32 min of annealing, the Rh 3d_5/2_ region shows a noticeable reduction in the intensity of the component at 306.9 eV, accompanied by the emergence of a new feature at approximately 306.5 eV. This new component begins to appear as early as 3–4 min into the annealing process, indicating the early onset of surface chemical or structural transformation. The observed decrease at 306.9 eV in the difference plot exceeds the corresponding increase at 306.5 eV, suggesting some Rh diffusion into bulk giving rise to a new chemical or structural environment. The rapid emergence of this component suggest alloying or intermixing with Pt atoms diffusing from the substrate.^36^

Prior to Rh deposition and heat treatment, Pt 4f XPS spectrum shows a 4f doublet with Pt 4f_7/2_ at 70.9 eV (SI, Section S7), confirming a metallic Pt surface.^37–39^ Similarly to Rh 3d, the in situ Pt 4f spectrum gathered during annealing at 700 K is shown in Figure 6a (experiment 2). An increase in the overall spectral intensity was observed, with the most substantial change occurring within the initial 6 min of the heat treatment. While the overall XPS spectra remains comparable for annealing beyond this duration, a closer look into the individual XPS sweeps during the initial 2.5 min showed a more rapid change (Figure 6b,c). In this initial period, the Pt 4f peaks shifted +0.1 eV and exhibited a marked increase in intensity on the left-hand shoulder of the Pt 4f_7/2_ peak.

These spectral changes are indicative of a significant restructuring in the near-surface region. The overall increase in intensity suggests Pt enrichment in the analyzed volume. Concurrently, the emergence of the new shoulder at 71.2 eV points to a new chemical state for Pt, consistent with alloying with Rh. These observations suggest that significant atomic rearrangement and intermixing take place during the initial stages of annealing, leading to a Pt-enriched surface. This restructuring can be the result of either segregation of Pt to the surface, diffusion of Rh into the subsurface region, or a combination of both processes, in line with reported work.^36^

Peak fitting of Pt 4f spectra (SI, Section S8) reveals a slight relative increase in the bulk-like Pt component upon annealing, alongside higher binding energy shifts of Pt 4f_7/2_ consistent with Pt–Rh intermixing.^36^ Due to the bulk-weighted information depth of laboratory Al Kα XPS (∼1–10 nm), surface core-level components remain weakly resolved,^40^ limiting precise quantification of surface Pt enrichment.

### 3.6 Atomic Diffusion of Pt and Rh

To confirm the Pt and Rh restructuring suggested from in situ XPS, we evaluated the feasibility of bulk Pt–Rh diffusion using literature-reported parameters^17,41^ and classical diffusion theory (SI, Section S9). Arrhenius analysis shows that bulk diffusion of Pt into Rh and Rh into Pt is negligible at 700 K in 32 min, excluding bulk diffusion as the dominant pathway over the time scale of the experiments. At 900 K for 24 min, however, the calculated diffusion depths of Pt diffusion into Rh and Rh diffusion into Pt to increase to 0.09 and 2.16 nm, respectively, making Rh diffusion significant. This enhanced intermixing could account for the breakdown of the long-range honeycomb ordered structure seen after 24 min at 900 K (Figure 3f), but not for the 6 min case (SI, Section S5).

Given the negligible bulk diffusion at 700 K, grain boundary (GB) diffusion, typically several orders of magnitude faster than bulk diffusion, is considered.^18^ Since no experimental GB diffusion coefficients are available for the Pt–Rh system, the value of Pt on Co at 350 °C (*D* ∼ 1.1 × 10^–13^ m^2^/s) is applied,^42^ estimating that <1 s (SI, Section S9) is required for Pt/Rh to traverse a Rh/Pt GB-layer of ∼2.46 nm (11.2 ML). Surface diffusion of Pt on Rh(111) at 700 K (*D* ∼ 10^–13^–10^–11^ m^2^/s; SI Section S9)^43^ enables rapid lateral redistribution (≪1 s) once Pt reaches the Rh surface.^44^ Thus, GB and surface diffusion likely dominate Pt–Rh transport to drive honeycomb network formation at 700 K, while bulk diffusion is negligible.

### 3.7 Proposed Mechanism for Long-Range Ordered Honeycomb Formation on Rh/Pt(111)

We interpret the honeycomb network as a strain-driven reconstruction of a Pt–Rh alloyed surface layer, similar to those reported for Pt_3_Sn­(111),^1^ Pt_25_Ni_75_(111),^9,10^ and CoSi_2_ on Si(111),^11^ arising from lattice mismatch in the topmost atomic layers. The formation of the honeycomb structure on Rh/Pt(111) is supported by STM, in situ XPS, LEED, and estimated Pt/Rh diffusion lengths. A schematic illustrating the proposed formation steps for a postdeposition annealing temperature of 700 K is shown in Figure 7.

**Figure 7:**
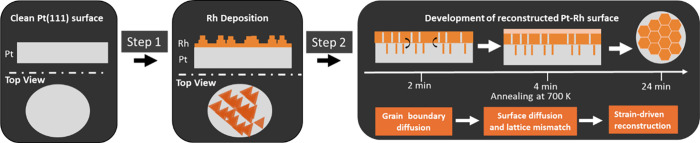
Schematic illustration of the proposed mechanism for honeycomb structure formation upon post deposition annealing Rh on Pt(111) at 700 K. Step 1, Rh deposition at 350 K forms densely packed triangular islands. Step 2, upon annealing at 700 K within the first few min, Pt atoms begin to diffuse from the Pt(111) substrate, primarily through the grain boundaries of the Rh islands, while bulk diffusion remains negligible. As Pt reaches the surface (Step 2), enhanced surface diffusion promotes uniform redistribution across the Rh overlayer. A Pt–Rh top layer forms above a Rh-rich layer, inducing lattice strain due to atomic mismatch. This strain drives surface reorganization into a honeycomb structure. With continued annealing limited bulk diffusion may occur at the interface.

In Step 1 (Figure 7), Rh deposition at 350 K on clean Pt(111) forms densely packed triangular Rh islands (Figure 1c–f), with some Pt either exposed between islands or diffused into Rh.^6,26^ Upon postdeposition annealing at 700 K (Step 2, Figure 7), islands merge and flatten, resulting in formation of the long-range ordered honeycomb pattern within minutes. In line with morphological changes, in situ XPS shows composition changes with annealing time: Rh 3d decreases as Rh become diluted with diffused Pt (Figure 5a), while Pt 4f intensity slightly increases (Figure 6b) as Pt diffuses via grain boundaries and across Rh surfaces.

Based on our estimates of Pt and Rh transport properties in bulk versus GBs or surfaces (Section 3.6), the transformation is dominated by GB and surface diffusion at 700 K. As Pt reaches the Rh surface (Step 2, Figure 7), the high atomic surface mobility of both Pt and Rh enables rapid elemental redistribution across the Rh overlayer. This results in the formation of a Pt-containing layer atop a Rh-rich subsurface. Evidence for Pt–Rh alloying in the top atomic layers is supported by in situ XPS measurements, where new Rh 3d and Pt 4f and species appear (Figures 5b and 6a) within the first 2 min of postdeposition annealing at 700 K. We note that these interpretations are drawn within the known probing depth limitations of laboratory XPS with Al Kα excitation, which yields bulk-weighted spectra and does not allow a clear distinction between the top atomic layers and Rh-rich sub surface. Taken together, the observed increase in Pt signal and the accompanying chemical shifts are consistent with Pt–Rh intermixing in the surface region. The Pt–Rh top layer has a slightly enlarged lattice relative to the underneath Rh rich layer due to the larger lattice constant of Pt (*a*_Pt_ = 3.92 Å^9^ and *a*_Rh_ = 3.80 Å^45^), creating interfacial strain similar to sputtered and annealed Pt_3_Sn­(111)^9^ and Pt_25_Ni_75_(111).^10^ This strain is relieved by slight atomic displacements relative to the underlaying lattice, resulting in the long-range honeycomb corrugation pattern as observed in STM (Figure 2c).

The model is also consistent with LEED observations: after 700 K annealing showing triangular composite spots (Figure 4c). Still, the overall symmetry of the pattern closely resembles that of Rh(111), suggesting Rh dominates the surface region probed by LEED (∼0.5–1 nm).^35^ This reflects the substantial 11.2 ML Rh coverage beneath the alloyed surface layer. Simultaneously, Pt diffusion during annealing modifies the top atomic layers, producing a mixed Pt–Rh surface. The transformation of the three main Rh LEED spots into triangular faceted spots may be a signature of the reconstruction.

The stability of the long-range honeycomb structure is governed by the interplay of annealing temperature and time. At 700 K, Rh bulk mobility is negligible (diffusion depth ≪ 0.01 nm after 32 min), and the honeycomb remains stable. In contrast, increasing the postdeposition annealing temperature to 900 K, Rh mobility increases markedly, reaching a diffusion depth of ∼2.16 nm after 24 min, leading to Pt–Rh intermixing that disrupts the Rh overlayer and destroys long-range order (Figure 3f). In contrast, a short 6 min anneal at 900 K still yields a stable honeycomb (SI, Section 5), comparable to 24 min at 700 K. This highlights the sensitive balance between temperature, annealing time, and Pt–Rh composition, and supports a strain-driven reconstruction rather than a moiré-type origin.

The proposed formation mechanism is of fundamental interest and could be further validated by depth-, photon energy-, and angle-resolved XPS at a soft X-ray synchrotron, which would allow the Pt–Rh compositional gradient to be resolved and the chemical nature of the top layer to be identified more clearly. Surface-sensitive X-ray diffraction could further clarify the long-range order, lattice registry, and possible in-plane distortions associated with the honeycomb reconstruction.^46^ Complementary DFT calculations would provide atomic-scale insight into alloying and strain-driven reconstruction, making these approaches promising directions for future work.

### 3.8 Extension of the Morphological Roadmap for Rh/Pt(111) and Perspectives for Catalysis

Our previous studies^5,6^ on Rh/Pt(111) and Pt/Rh(111) surfaces have established a roadmap for the evolution of surface structures at low Rh- and Pt-coverages (<1 ML), respectively, and annealing temperatures (450–900 K) (see SI, Section S10). In brief, a range of surface morphologies, including triangles, hexagons, worm-like features, and domains were discovered, with each morphology influenced by specific process parameters such as substrate, the presence of surface impurities,^47,48^ and the deposition rate/flux, particularly at submonolayer coverages. The current study extends this roadmap into the high Rh-coverage regime (up to 11.2 ML Rh) (Figure S10, left panel; SI, Section S10). At 300–400 K, Rh triangles deposited on Pt(111) are the dominate surface features, which convert by postdeposition annealing at 700 K to a well-ordered hexagonal honeycomb network across the surface. The onset of reconstruction formation coincides with increased Pt diffusion onto the surface at 500 K, creating isolated domains of the reconstruction. At 900 K, the honeycomb structure can still form, but only when the annealing time is sufficiently short (e.g., 6 min, SI, Section 5). Limited Pt–Rh intermixing allows the honeycomb reconstruction to develop before significant bulk alloying takes place. Longer annealing at 900 K produces a less regular and increasingly distorted pattern due to enhanced bulk diffusion (Figure 3f). This behavior defines a stability window for the reconstruction centered around 700 K, with formation at 900 K being strongly time restricted.

The roadmap for tailoring Pt–Rh surfaces (Figure S10) provides an excellent guide for producing well-defined model surface catalysts for systematic investigations to correlate catalytic performance with surface alloying. Catalytic behavior is highly alloy composition dependent. For example, in the NH_3_ slip reaction over Pt–Rh alloys, Pt-rich surfaces are significantly more active toward NH_3_ conversion than Rh surfaces. The product distribution (NO versus N_2_) is governed by the surface concentration of adsorbed N and O species. These concentrations are strongly temperature dependent and sensitive to the gas-phase environment (O_2_, NH_3_, H_2_O, etc.). They also depend on which of the two alloy constituents (Pt or Rh) that predominates the surface.^4^ The honeycomb pattern discovered in this study, likely composed of Pt enriched Rh surface, could serve as an excellent model catalyst for exploring next-generation Pt–Rh alloys for NH_3_ slip oxidation. We propose research along this axis would be a fruitful avenue for systematic fundamental studies aimed at optimizing Pt–Rh alloying to enhance both catalytic activity and selectivity toward N_2_ for the NH_3_ slip reaction, or for studies of other relevant oxidation processes.

## 4 Conclusions

This study reports the formation of a long-range ordered honeycomb surface reconstruction upon thermal annealing of Rh multilayers deposited on Pt(111) at 350 K. The reconstruction consists of well-defined hexagonal patterns with a characteristic diagonal length of approximately 15 ± 2 nm and a height corrugation of ∼0.07 nm. It develops most uniformly for relatively high Rh coverages (on the order of ∼11.2 ML) and annealing temperatures around 700 K. STM imaging shows that at these conditions the surface exhibits a highly ordered and continuous honeycomb network. Annealing at 500 K initiates only partial reconstruction, producing isolated domains, while annealing at 900 K gives regular honeycombs for short annealing times (6 min) but leads to irregular or disrupted structures for longer annealing times due to enhanced Rh–Pt intermixing.

These findings establish that the honeycomb reconstruction is stabilized by a delicate interplay of Rh-coverage, annealing temperature, and time and the surface alloying/intermixing behavior of Pt and Rh.

Our analysis shows that, at 700 K, surface and grain boundary diffusion processes are the primary mechanisms driving Pt–Rh intermixing, while bulk diffusion of Pt and Rh atoms is negligible over the experimental time frame due to extremely low diffusivity. This contrasts sharply with behavior at 900 K for longer durations, where higher bulk diffusion enables significant atomic transport and deep intermixing, fundamentally altering the surface morphology.

In summary, for the 700 K scenario, Pt atoms diffuse to the Rh surface, and their high surface mobility facilitates rapid redistribution across the Rh overlayer, resulting in the formation of a Pt–Rh top layer above a Rh-rich subsurface. The resulting lattice mismatch between the Pt–Rh top layer and the underlying Rh-rich layer introduces significant interfacial strain. Continued annealing enables strain relaxation through surface reorganization, ultimately driving the formation of the observed honeycomb network. These results highlight the critical role of directional atomic diffusion and strain-driven reconstruction in the self-assembly of complex surface nanostructures in bimetallic systems.

## Supplementary Material



## Notes

Section 1: Surface cleaning procedure, removal of Rh deposited on Pt(111) after 7 cycles of cleaning. Section 2: Surface coverage calculations. Section 3: Overview of the honeycomb dimensions as a function of postdeposition annealing time. Section 4: Effect of postdeposition annealing time on formation of honeycomb structure. Section 5: Postdeposition annealing of 11.2 ML Rh/Pt(111) at 900 K for 6 min. Section 6: Fast Fourier transform (FFT) and moiré periodicity analysis. Section 7: Pt peak position of a clean surface. Section 8: XPS peak fitting analysis. Section 9: Diffusion model parameters and calculations. Section 10: Pt–Rh Roadmap (PDF)
