# Supplementary material for: Strain-Driven Honeycomb Reconstruction of Multilayered Rh/Pt(111)
Source: ACS Appl Mater Interfaces. 2026 May 25;18(21):30446–55. doi: 10.1021/acsami.5c25647 (PMC13244363; doi:10.1021/acsami.5c25647)
Supplement: Supplementary file 1 [file am5c25647_si_001.pdf]

Supplementary information

Strain-Driven Honeycomb Reconstruction of Multilayered Rh/Pt(111)

Abdulla Bin Afif[1,*], Oleksii Ivashenko[1,2], Alexandra Jahr Kolstad[1] and Anja Olafsen Sjåstad[1,*]

[1]Centre for Materials Science and Nanotechnology, Department of Chemistry, University of Oslo, P.O. Box 1033 Blindern, N-0315 Oslo, Norway
[2]DNV AS, Veritasveien 1, N-1363 Høvik, Norway

*Correspondence: a.b.afif@kjemi.uio.no and a.o.sjastad@kjemi.uio.no

<u>Section S1</u>: Surface cleaning procedure, removal of Rh deposited on Pt(111) after 7 cycles of cleaning

Each cycle consisted of sputtering of Ar^+ ions at 1 kV and a pressure of 1.1×10^{-5} mbar for 10 min, followed by annealing at 1100 K under UHV for 10 min.

No Rh was detected using XPS on the Pt(111) surface after the cleaning procedure.

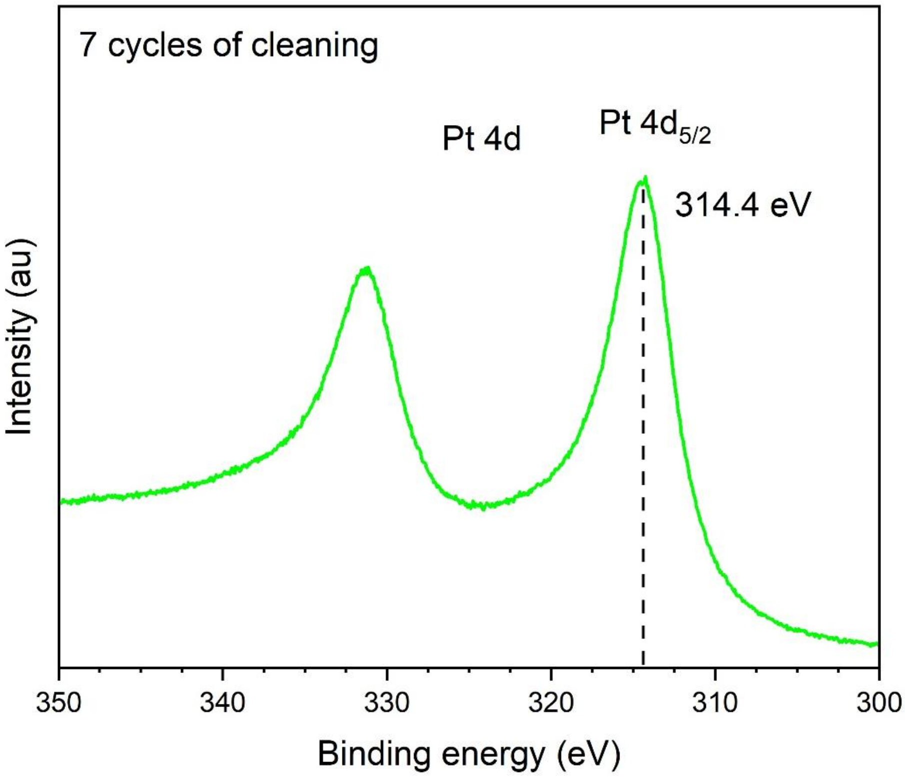

Figure S 1: XPS Pt 4d core level spectrum recorded after seven cycles of Ar^+ sputtering and annealing of a Pt(111) surface, showing clear Pt $4d_{5/2}$ at 314.4 eV, and absence of Rh $3d_{5/2}$ at 307 eV, confirming that seven cleaning cycles are sufficient to remove Rh from the surface.

Section S2: Surface coverage calculations

Rh surface coverage was determined by segmenting and masking the STM topography image into height-defined layers in the Gwyddion software and summing their projected areas. The coverage for the low-coverage sample (1.4 ML Rh after 4 min deposition) was first determined directly from STM analysis and used to calibrate the evaporation rate. This calibrated rate was then applied to estimate the coverages of thicker films, where island coalescence made direct measurements less reliable.

1. Scan area: 97 nm × 91 nm = 8 853 nm²
2. Layer projections with 0.21 nm thresholds:
   - Layer 1: 6 874 nm²
   - Layer 2: 4 684 nm²
   - Layer 3: 763 nm²
   - Total projected area: 6 874 + 4 684 + 763 = 12 321 nm²
3. Coverage calculation:

$$\text{Coverage (ML)} = \frac{\text{Total projected area}}{\text{Scan area}} = \frac{12\,321\ \text{nm}^2}{8\,853\ \text{nm}^2} \approx 1.39\ \text{ML}$$

Hence, the Rh deposition corresponds to a surface coverage of approximately 1.4 ML.

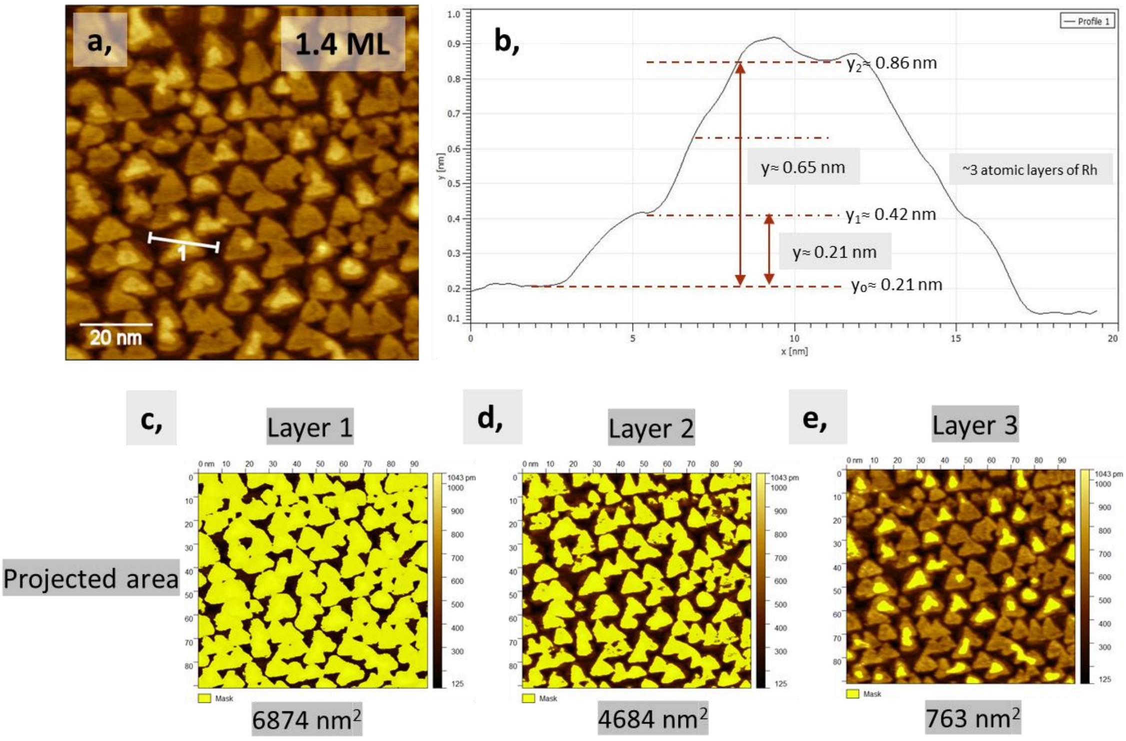

Figure S 2: Surface-coverage determination by height-threshold segmentation of the STM data. a) Original STM topography of Pt(111) after 4 min Rh deposition (flux 8 nA). b) Representative line profile across a Rh island, showing step heights of ~0.21 nm (one atomic layer), ~0.42 nm (two layers) and ~0.65 nm (three layers). Horizontal dashed lines mark the height thresholds used to segment layers 1-3. (c-e) Binary masks of the projected area occupied by layers 1, 2 and 3, respectively. Dividing the total projected area by the scan area (8 853 nm²) yields a surface coverage of ~1.4 ML.

<u>Section S3</u>: Overview of the honeycomb dimensions as function of post-deposition annealing time

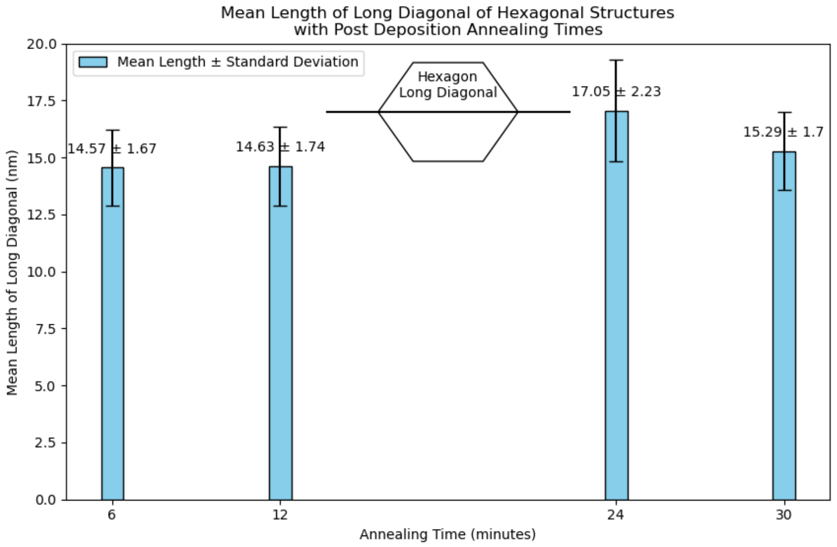

Figure S 3: Mean diagonal length of the hexagonal cells as a function of post-deposition annealing time at 700 K. Each bar represents different annealing times, with error bars indicating one standard deviation. The diagonal remains in the same range 14.6 ± 1.7 nm at 6 min, 14.6 ± 1.7 nm at 12 min, 17.1 ± 2.2 nm at 24 min and 15.3 ± 1.7 nm at 30 min, demonstrating that extended annealing does not significantly alter the size of the honeycomb network. For each annealing time, long-diagonal length of ~100 honeycombs are measured using ImageJ software[1].

Section S4: Effect of post-deposition annealing time on formation of honeycomb structure

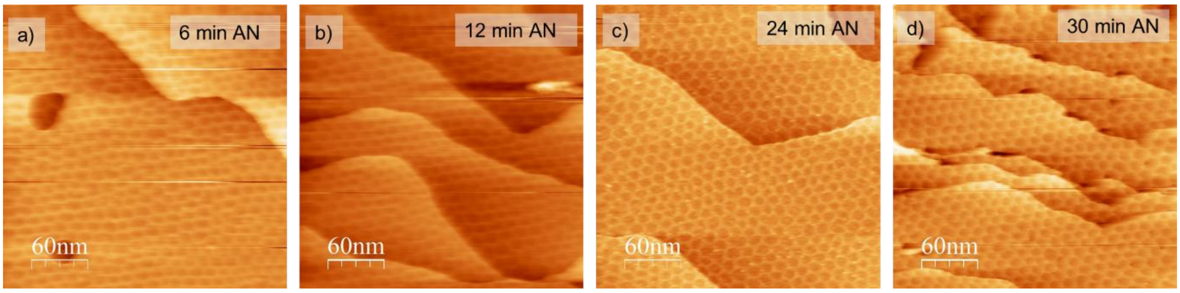

Figure S 4: STM images of 11.2 ML Rh-covered Pt(111) following annealing at 700 K for a) 6 min, b) 12 min, c) 24 min and d) 30 min. All images (60 nm scale bar) show the characteristic honeycomb network forming by 6 min and persisting with unchanged cell size and shape through 30 min.

<u>Section S5</u>: Post-deposition annealing of 11.2 ML Rh/Pt(111) at 900 K for 6 min

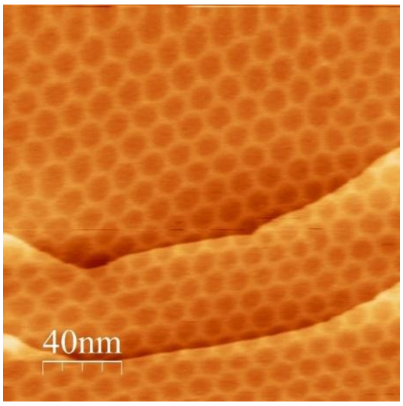

Figure S 5: STM image of Rh/Pt(111) surfaces after deposition of 11.2 ML Rh at 350 K, followed by post-deposition annealing for 6 min at 900 K.

Section S6: Fast Fourier transform (FFT) and moiré periodicity analysis

S6.1 FFT Analysis

Experimental versus simulated moiré FFTs

We analyzed the FFT of the experimentally observed honeycomb pattern (Figure S 5) formed after annealing at 900 K for 6 min and compared it with a simulated FFT of a relaxed Pt(111) overlayer on Rh(111), using lattice constants of $a_{Pt(111)}$ = 3.99 Å[2] and $a_{Rh(111)}$ = 3.80 Å[3]. The simulations were performed using PyAtoms.[4]

The FFT of the honeycomb pattern exhibits a pronounced streak through the origin, indicating a strong one-dimensional component or anisotropy, consistent with step bunching, terrace structures, or scan-direction-related features. The intensity maxima near the center are elongated and distributed along this streak, rather than forming a single, small, symmetric hexagon. This behavior points to anisotropic and possibly overlapping periodicities, rather than a single, well-defined two-dimensional superlattice.

The FFT therefore indicates the presence of long-range order with a characteristic length scale in the tens of nanometers, but it does not support a simple geometric moiré interpretation. For comparison, we also include simulated real-space images and corresponding FFTs for Rh(111) on Pt(111) and for twisted graphene on graphene, which serve as benchmark system exhibiting well-defined moiré signatures. Clear differences are observed between these reference FFTs and those obtained for the present Rh/Pt(111) system.

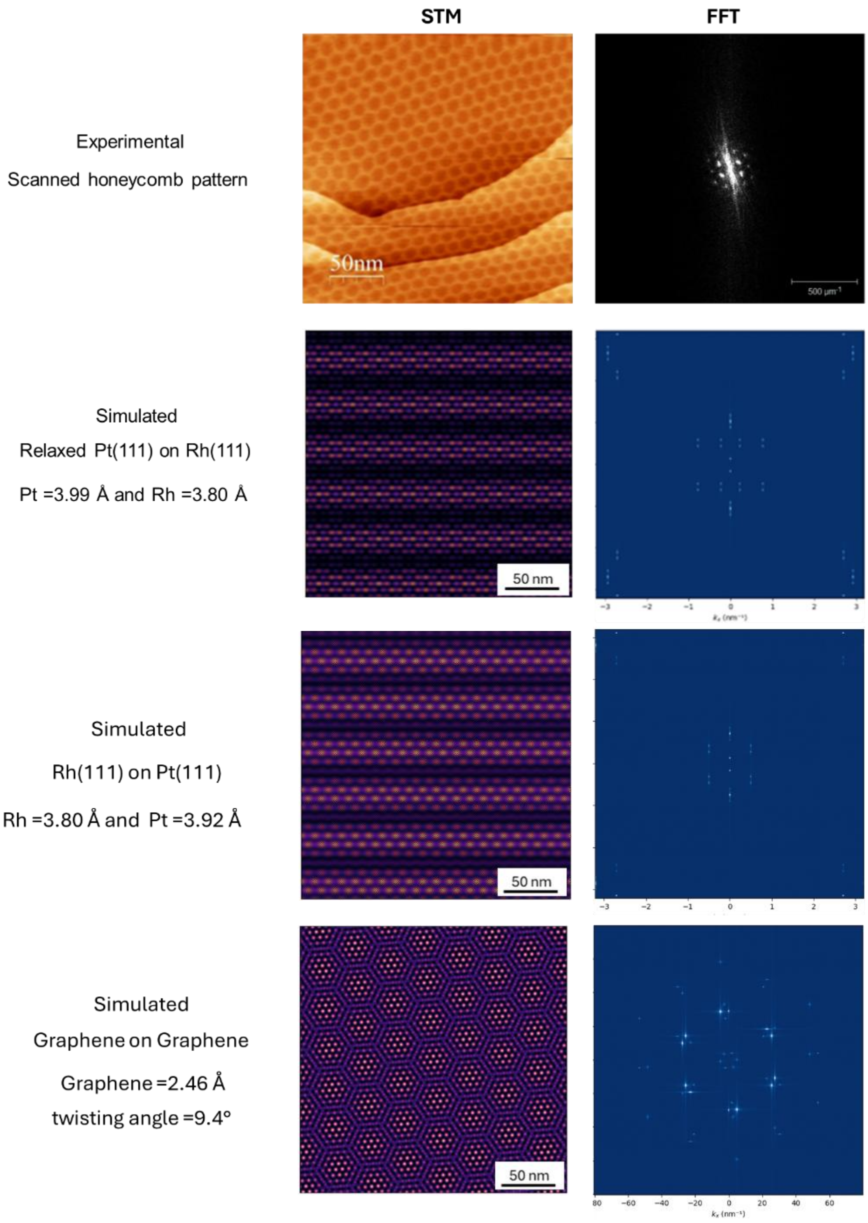

Figure S 6: Real-space STM and FFT comparison distinguishing honeycomb reconstruction from geometric moiré. (a-b) Experimental STM image of honeycomb reconstruction (900 K, 6 min annealing) and its FFT, showing anisotropic central streak with elongated intensity maxima. (c-d) Simulated Pt(111)/Rh(111) moiré (a_{Pt} = 3.99 Å[2], a_{Rh} = 3.80 Å[3]), exhibiting discrete symmetric hexagon spots characteristic of rigid-lattice moiré. (e-f) Simulated Rh(111)/Pt(111) moiré (a_{Rh} = 3.80 Å[3], a_{Pt} = 3.92 Å[5]), showing well-defined hexagonal FFT pattern. (g-h) Twisted graphene bilayer (~2.4° twist) benchmark, displaying classic six-spot moiré FFT signature.

S6.2 Moiré periodicity analysis

We calculate the moiré length resulting from the superposition of a relaxed Pt(111) lattice on a Rh(111) lattice using nearest-neighbor distances.
For a fully relaxed Pt(111) surface, we adopt a lattice parameter of 3.99 Å[2], while the underlying Rh-rich layer is described by a lattice parameter of 3.80 Å[3]. These values correspond to nearest-neighbor distances of approximately 2.82 Å for Pt(111) and 2.69 Å for Rh(111). Using the standard expression for hexagonal lattices, the resulting moiré periodicity is approximately 10 nm. This value is smaller than the experimentally observed honeycomb periodicity of about 15 nm.

$$L_{Moire} = \frac{a_{Rh} \cdot a_{Pt}}{|a_{Rh} - a_{Pt}|} \cdot \sqrt{3} \quad \text{- Eq. (S1)}$$

$$L_{Moire} \approx 10.01 \text{ nm}$$

We further evaluated additional scenarios to assess the sensitivity of the moiré length to lattice parameter variations. Using the bulk Pt lattice constant (*a* = 3.92 Å[5]) together with Rh (*a* = 3.80 Å) yields a moiré periodicity close to 15 nm, while assuming a reduced Pt lattice parameter representative of a Pt-Rh alloyed surface (e.g., 3.85 Å) results in a larger moiré length, on the order of 40 nm. The comparison shows that a simple geometric moiré pattern formed by a relaxed Pt overlayer cannot consistently account for the observed periodicity. Instead, the experimentally measured value lies between the limiting cases of a fully relaxed Pt layer and a compressed alloyed overlayer. Based on this analysis, we favor a strain-driven reconstruction mechanism rather than a purely geometric moiré overlayer, for several reasons, Firstly, in-situ XPS indicates that the surface is not pure Pt but a Pt-Rh alloy, which would, according to Vegard-type arguments, reduce the effective surface lattice parameter and increase the expected moiré length beyond the observed values with pure Pt(111).
Secondly, the reconstruction exhibits a pronounced dependence on Rh thickness and annealing temperature, forming only above a critical coverage and degrading when annealed at 900 K for longer duration (24 min) due to enhanced bulk diffusion. Such behavior is characteristic of strain-relief reconstructions.
Finally, the continuity of the honeycomb structure across step edges further supports a reconstruction mechanism rather than a simple moiré interference effect.

Moiré pattern simulations: Comparative lattice constant analysis

To validate the theoretical periodicity of the moiré pattern, simulations were performed across a range of lattice constants.
The simulations were carried out using two different programs to ensure computational consistency and cross-verify the results:

1. PyAtoms[4]
2. NanoHUB (moiré calculator)[6]

The results from both simulators showed agreement, yielding identical patterns. The representative patterns generated via PyAtoms are illustrated below:

<u>Case 1</u>: Relaxed Pt(111) on Rh(111)

Lattice constants: a_{Pt} = 3.99 Å[2] and a_{Rh} = 3.80 Å[3]

The lattice constant for the overlayer was adjusted to account for surface relaxation effects, which differentiate the surface atomic spacing from bulk values. Experimental measurements indicate an outward expansion of the first two layers of Pt(111)[7], effectively increasing the lattice constant to approximately 3.98 Å. This can be supported by theoretical study that report an increase from 3.92 Å to a relaxed surface value of 3.99 Å.[2] Consequently, a value of 3.99 Å was adopted for the relaxed Case 1 calculation.

Calculated moiré length: 10.1 nm

<u>Case 2</u>: Lattice constant of Pt(111) with bulk value

Lattice constants: a_{Pt} = 3.92 Å[5] and a_{Rh} = 3.80 Å

Calculated moiré length: 15 nm

<u>Case 3</u>: Alloying and lattice modulation

Lattice constants: a_{Pt} = 3.85 Å and a_{Rh} =3.80 Å

Under the assumption that the overlayer is a surface alloy rather than pure Pt, the lattice parameters must be adjusted to account for the chemical composition. According to Vegard's Law, the lattice constant of an alloy is a weighted average of its constituents; therefore, the incorporation of Rh into the Pt lattice induces a linear contraction of the overlayer lattice constant.

Calculated moiré length: 42.4 nm

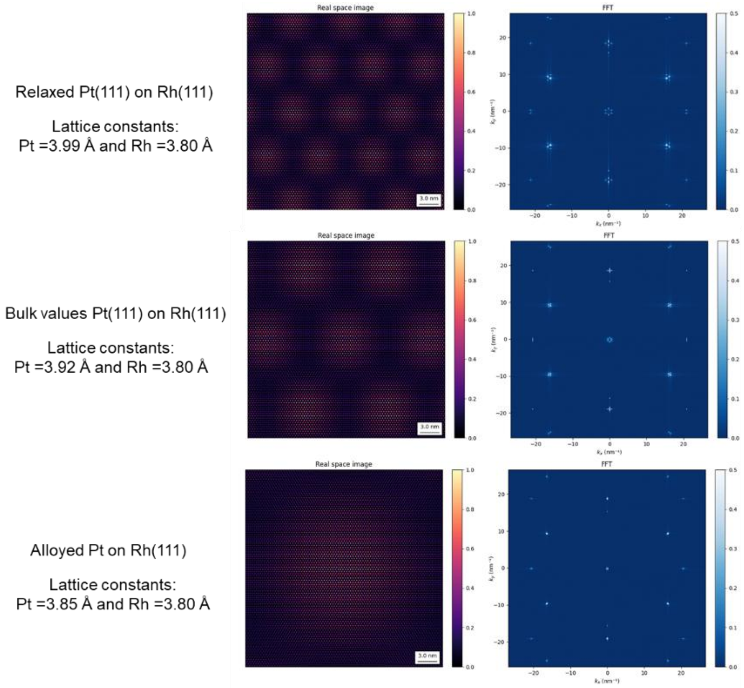

Figure S 7: Moiré superlattice of different cases from top to bottom. Left: Real-space image and Right: Corresponding Fast Fourier Transform (FFT).

<u>Section S7</u>: Pt peak position of a clean surface

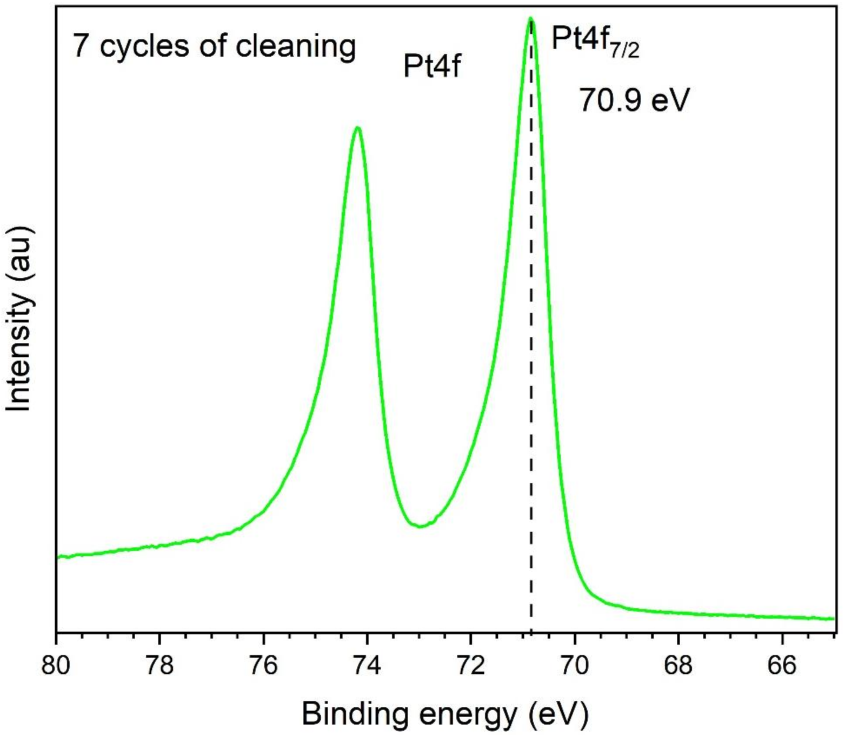

Figure S 8: XPS spectrum of the Pt 4f region acquired on Pt(111) after seven sputter/anneal cleaning cycles. The spin-orbit doublet is clearly resolved, with the Pt 4f$_{7/2}$ peak located at 70.9 eV (vertical dashed line).

Section S8: XPS peak fitting analysis

Evaluating the Pt 4f bulk and surface components at the start and after 32 min of post-deposition annealing

Pt 4f spectra is fitted using a Shirley background and a constrained doublet. The Pt 4f region is described by two spin-orbit doublets, attributed to bulk-like and surface-like Pt, respectively. For each doublet, the components are constrained to a fixed spin-orbit splitting of 3.33 eV, with an area ratio of 4:3. The bulk-related Pt $4f_{7/2}$ component is fixed at 70.86 eV and the corresponding Pt $4f_{5/2}$ at 74.19 eV, whereas the surface-related Pt $4f_{7/2}$ and Pt $4f_{5/2}$ components are positioned at 70.40 and 73.73 eV, respectively. All peaks were fitted using a mixed Gaussian-Lorentzian line shape.

Closer inspection of Pt 4f fits indicates that the bulk-like component slightly increases upon annealing in comparison to surface species. This behavior suggests that the annealing treatment does not simply convert bulk Pt into a predominantly surface-like state but rather leads to a situation where a larger fraction of the detected Pt signal remains in, or is redistributed into, a more bulk-like chemical environment (for example, Pt-Rh alloy region). Consequently, these spectra should be interpreted with caution in the context of “surface enrichment”: while subtle changes in the surface-related component are present, the visible effect captured by this deconvolution is an increase of the bulk contribution, indicating that the structural and compositional evolution during annealing is more complex than a straightforward buildup of a thin Pt-enriched surface layer.

It is important to note, that the laboratory Al Kα source (hv = 1486.6 eV) used in this study produces bulk-weighted spectra with limited sensitivity to the outermost atomic layer. With the present instrumentation, any surface component is too weak to be clearly distinguished from bulk shoulders or fitting artifacts, ruling out reliable quantification of surface Pt enrichment solely from this analysis.

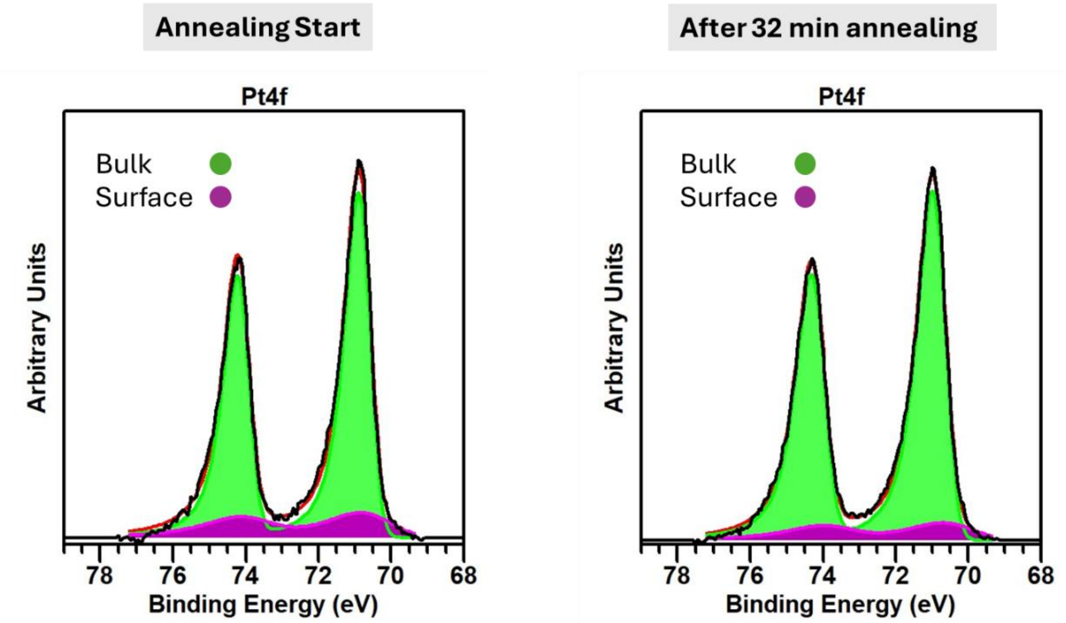

Figure S 9: Pt 4f peak fitting before and after annealing. Deconvolution of Pt 4f spectra at the start of annealing (left) and after 32 min at 700 K (right), using two spin-orbit doublets assigned to bulk-like (green) and surface-like (magenta) Pt components. The fits show a slight increase of the bulk-like contribution and an overall broadening/shift of the Pt 4f envelope after annealing, consistent with Pt diffusion into a Pt-Rh near-surface alloy while the spectra remain dominated by bulk-weighted signal due to the Al Kα excitation energy.

<u>Section S9</u>: Diffusion model parameters and calculations

To evaluate atomic diffusion in the Rh/Pt(111) system, we employed the Arrhenius relation [8]:

$$D(T) = D_0 \cdot \exp\left(-\frac{E_a}{k_B T}\right) \text{ - Eq. (S2)}$$

Where:

- $D(T)$ is the diffusion coefficient at temperature T (K)
- D_0 is the pre-exponential factor, diffusion coefficient in absence of energy barriers
- E_a is the activation energy for diffusion
- k_B is Boltzmann's constant (8.617 × 10^{-5} eV/K)

1. Bulk diffusion of Pt into Rh

1.1 Bulk diffusion at 700 K

From literature, the bulk diffusion coefficient for Pt into Rh is:

- $D = 1.6 \times 10^{-19}$ m^2/s at 1273 K [9]
- $E_a = 3.15$ eV

Using this value and solving the Arrhenius equation for two temperatures 700 K and 1273 K:

$$D(T_2) = D(T_1) \exp\left[-\frac{E_a}{k_B}\left(\frac{1}{T_2} - \frac{1}{T_1}\right)\right] \quad \text{ - Eq. (S3)}$$

- $D_{Pt \to Rh}(700\,K) \quad = 1.6 \times 10^{-19} \exp\left[-\frac{3.15}{8.617\times10^{-5}}\left(\frac{1}{700} - \frac{1}{1273}\right)\right]$
  $\approx 9.9 \times 10^{-30}\ m^2\, s^{-1}$.

The diffusion depth over t= 32 minutes (1920 seconds) is calculated using:

$$x = 2.4 \cdot \sqrt{Dt} \text{ - Eq. (S3)}$$

$$x \approx 0.0003308\,\text{nm}$$

1.2 Bulk diffusion at 900 K for 24 minutes

Using this value and solving the Arrhenius equation for two temperatures 900 K and 1273 K:

$$D(T_2) = D(T_1) \exp\left[-\frac{E_a}{k_B}\left(\frac{1}{T_2} - \frac{1}{T_1}\right)\right]$$

- $D_{Pt \to Rh}(900\,K) \quad = 1.6 \times 10^{-19} \exp\left[-\frac{3.15}{8.617\times10^{-5}}\left(\frac{1}{900} - \frac{1}{1273}\right)\right]$
  $\approx 1.08 \times 10^{-24}\ m^2\, s^{-1}$.

The diffusion depth over t = 24 minutes (1440 seconds) is calculated using:

$$x = 2.4 \cdot \sqrt{Dt} \Rightarrow x \approx 0.09487\ nm$$

2. Bulk diffusion of Rh into Pt

2.1 Bulk diffusion at 700 K

From literature, the bulk diffusion coefficient for Rh into Pt is:

- $D = 1.6 \times 10^{-22}$ m^2/s at 873 K [8]

Using this value and solving the Arrhenius equation for two temperatures 873 K and 700 K:

$$D(T_2) = D(T_1)\exp\left[-\frac{E_a}{k_B}\left(\frac{1}{T_2} - \frac{1}{T_1}\right)\right]$$

- $D_{Rh \to Pt}(700\,K) \quad = 1.6 \times 10^{-22} \exp\left[-\frac{3.15}{8.617 \times 10^{-5}}\left(\frac{1}{700} - \frac{1}{873}\right)\right]$
  $\approx 5.12 \times 10^{-27}\ m^2\ s^{-1}$.

The diffusion depth over t = 32 minutes (1920 seconds) is calculated using:

$$x = 2.4 \cdot \sqrt{Dt} \Rightarrow x \approx 0.007529\,\text{nm}$$

2.2 Bulk diffusion at 900 K

From literature, the bulk diffusion coefficient for Rh into Pt is:

- $D = 1.6 \times 10^{-22}\ \text{m}^2/\text{s}$ at 873 K [8]

Using this value and solving the Arrhenius equation for two temperatures 873 K and 900 K:

$$D(T_2) = D(T_1)\exp\left[-\frac{E_a}{k_B}\left(\frac{1}{T_2} - \frac{1}{T_1}\right)\right]$$

- $D_{Rh \to Pt}(900\,K) \quad = 1.6 \times 10^{-22} \exp\left[-\frac{3.15}{8.617 \times 10^{-5}}\left(\frac{1}{900} - \frac{1}{873}\right)\right]$
  $\approx 5.62 \times 10^{-22}\ m^2\ s^{-1}$.

The diffusion depth over t= 24 minutes (1440 seconds) is calculated using:

$$x = 2.4 \cdot \sqrt{Dt} \Rightarrow x \approx 2.16\,\text{nm}$$

3. Grain boundary diffusion of Pt

No direct literature data for Pt in Rh grain boundaries was found. A conservative estimate of $D \approx 1 \times 10^{-17}\ \text{m}^2/\text{s}$ was used based on values from Pt in Co grain boundaries at 623 K [10].

The estimated diffusion time across 4 nm:

$$t = \frac{x^2}{4D} = \frac{(4 \times 10^{-9})^2}{4 \cdot 10^{-17}} \approx 0.28\,\text{s}$$

4. Surface diffusion of Pt on Rh(111)

Based on ref. [11], the activation energy for Pt surface diffusion on Rh(111) is below 0.22 eV. A conservative value of $E_a \approx 0.11\,\text{eV}$ was used with pre-exponential factors of $D_0 \approx 10^{-3}$ to $10^{-2}\ \text{cm}^2/\text{s}$, yielding:

$$D_{700K}\ \text{ranges from}\ 10^{-13}\ \text{to}\ 10^{-11}\ \text{m}^2/\text{s}$$

This indicates efficient lateral diffusion once Pt reaches the Rh surface.

Section S10: Pt-Rh Roadmap

Our previous studies[12, 13] on Rh/Pt(111) and Pt/Rh(111) surfaces have established a roadmap for the evolution of surface structures at low Rh- and Pt-coverages (< 1 ML), respectively, and annealing temperatures (450-900 K). In brief, a range of surface morphologies, including triangles, hexagons, worm-like features, and domains were discovered, with each morphology influenced by specific process parameters such as, substrate temperature for both as-prepared and post-annealed surfaces, the presence of surface impurities,[14] and the deposition rate/flux, particularly at sub-monolayer coverages. For Pt deposited on Rh(111), a lamellar ad-island reconstruction was observed, driven by lattice strain due to the size mismatch between the larger Pt lattice and the smaller Rh substrate (a_{Pt} = 3.92[5] and a_{Rh} = 3.80 Å[3]). Conversely, Rh deposition on Pt(111) resulted in network reconstructions, where small nuclei initially formed along the crystallographic symmetry directions. These reconstructions were believed to alleviate surface strain, with structural formations aligning with preexisting strain directions, acting as preferred nucleation sites. The STM and XPS analyses revealed the progression from isolated Rh ad-islands to surface alloy formation and eventually to near-surface alloy configurations. Deposition at room temperature followed by post-annealing above 550 K results in the transformation of triangular Rh islands into hexagonal structures, accompanied by increasing Pt-Rh intermixing, particularly at steps and terraces.

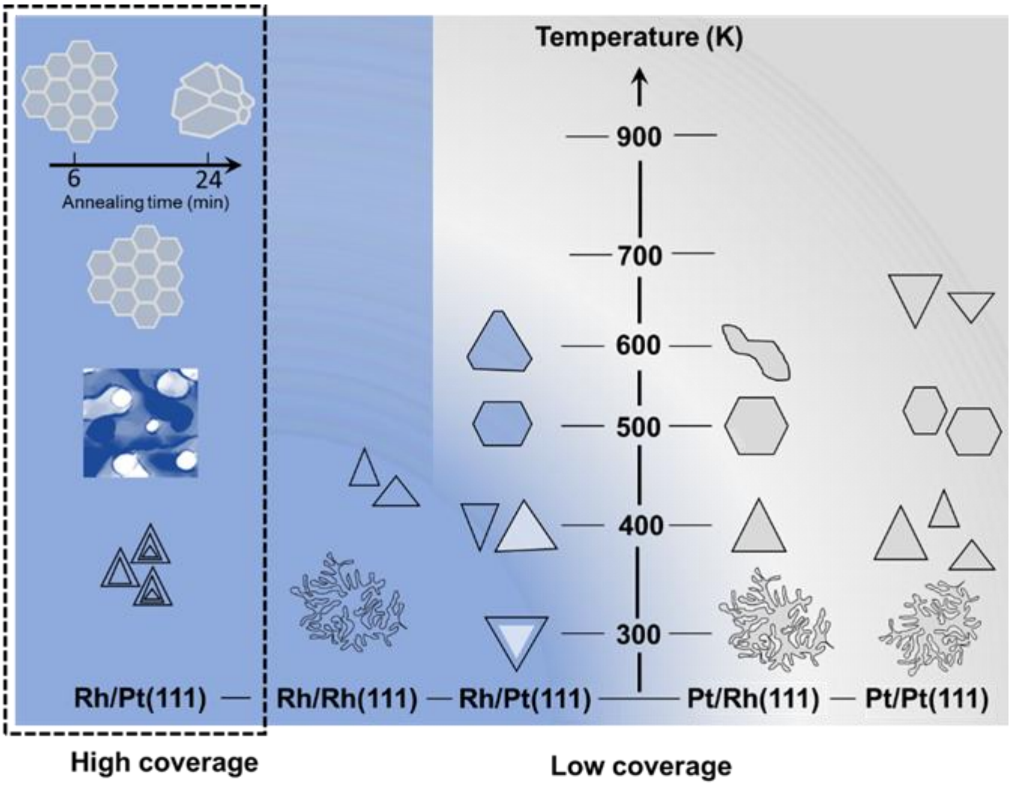

Figure S 10: Extended roadmap based on reference[12,13] of Rh/Pt(111) surface morphologies as a function of annealing temperature and Rh coverage. The new part of the diagram (within the dotted-line box) summarizes the morphological evolution observed upon annealing Rh films (11.2 ML) deposited on Pt(111) at 350 K. At 500 K, the surface exhibits predominantly worm-like features with irregular patches of honeycomb motifs. A well-ordered honeycomb network forms at 700 K (24 min). At 900 K the formation is time-dependent: brief annealing (e.g., 6 min) results in a regular honeycomb structure, while extended annealing (e.g., 24 min) produces a honeycomb structure with increased distortion due to enhanced bulk intermixing.

References

(1) Collins, T. J. ImageJ for Microscopy. *BioTechniques*. 2007, *43*, 25-30.
(2) Ichiya, T.; Koiwa, N.; Ohma, A.; Tada, S.; Fushinobu, K.; Okazaki, K. Surface Electronic: Atomic Structure and Activation Energy on Pt(111), Pt3Cu(111), and PtCu(111) for PEFC Cathode. *Nanoscale and Microscale Thermophysical Engineering*. 2010-4-30, *14* (2).
(3) Hermann, K. Appendix E: Parameter Tables of Crystals. In *Crystallography and Surface Structure*, Wiley-VCH, 2011; pp 265 -266.
(4) Prado, A. G.; Mandigo-Stoba, M. I.; Wey, K.-Y.; Nekarae, S.; Enriquez-Ibarra, A.; Bañuelos, S.; Nguyen, A.; Gutiérrez, C.; Prado, A. G.; Mandigo-Stoba, M. I.; et al. PyAtoms: An interactive tool for simulating atomic scanning tunneling microscopy images of 2D materials, moiré systems and superlattices. *arXiv e-prints*. 12/2024.
(5) Kuntze, J.; Speller, S.; Heiland, W.; Atrei, A.; Spolveri, I.; Bardi, U. Reconstruction and dislocation network formation of the (111) surface of the ordered alloy Pt_3Sn. *Physical Review B*. 1998, *58* (24), 16005-16008.
(6) Madhavan, K.; Zentner, L.; Farnsworth, V.; Shivarajapura, S.; Zentner, M.; Denny, N.; Klimeck, G.; Madhavan, K.; Zentner, L.; Farnsworth, V.; et al. nanoHUB.org: cloud-based services for nanoscale modeling, simulation, and education. *Nanotechnology Reviews*. 2013-02-01, *2* (1).
(7) Veen, J. F. V. D.; Smeenk, R. G.; Tromp, R. M.; Saris, F. W. Relaxation effects and thermal vibrations in a Pt(111) surface measured by medium energy ion scattering. *Surface Science*. 1979/01/01, *79* (1).
(8) Li, T.; Marquis, E. A.; Bagot, P. A. J.; Tsang, S. C.; Smith, G. D. W. Characterization of oxidation and reduction of a platinum–rhodium alloy by atom-probe tomography. *Catalysis Today*. 2011, *175* (1), 552-557.
(9) Kumar, S.; Waller, D.; Fjellvåg, H.; Sjåstad, A. O. Development of custom made bimetallic alloy model systems based on platinum – rhodium for heterogeneous catalysis. *Journal of Alloys and Compounds*. 2019, *786*, 1021-1029.
(10) Pedan, R.; Makushko, P.; Yavorskyi, Y.; Dubikovskyi, O.; Bodnaruk, A.; Burmak, A.; Golub, V.; Voloshko, S.; Hübner, R.; Makarov, D.; et al. Low-temperature diffusion in thin-film Pt-(Au-)-Co heterostructures: a structural and magnetic characterization. *Nanotechnology*. 2024, *35* (19).
(11) Kellogg, G. L. Diffusion of individual Pt atoms on single-crystal surfaces of rhodium. *Physical Review B*. 1993, *48* (15), 11305-11312.
(12) Pettersen, C.; Sjåstad, A. O.; Ivashenko, O. Near-Surface Alloys of PtRh on Rh(111) and Pt(111) Characterized by STM. *The Journal of Physical Chemistry C*. 2021, *125* (45), 25140-25147.
(13) Zheng, J.; Ivashenko, O.; Fjellvåg, H.; Groot, I. M. N.; Sjåstad, A. O. Roadmap for Modeling RhPt/Pt(111) Catalytic Surfaces. *The Journal of Physical Chemistry C*. 2018, *122* (46), 26430-26437.
(14) Kalff, M.; Comsa, G.; Michely, T. How Sensitive is Epitaxial Growth to Adsorbates? *Physical Review Letters*. 1998-08-10, *81* (6).
